# Right gluteal capillary hemangioma: a rare clinical image

**DOI:** 10.11604/pamj.2022.41.214.33862

**Published:** 2022-03-15

**Authors:** Mayur Bhaskar Wanjari, Tejaswee Lohakare

**Affiliations:** 1 Department of Research and Development, Jawaharlal Nehru Medical College, Datta Meghe Institute of Medical Sciences, Sawangi, Wardha, Maharashtra, India,; 2 Department of Child Health Nursing, Srimati Radhikabai Meghe Memorial College of Nursing, Datta Meghe Institute of Medical Sciences, Sawangi, Wardha, Maharashtra, India

**Keywords:** Lesion, angiofibrolipoma, right gluteal region

## Image in medicine

A case of an eight-year-old girl came to the emergency department with the complaint of a lesion on her right gluteal region with pain over the lesions from the last two months, which was about 5.1 × 10.7 × 13 cm in size at the onset. No one has a similar complaint about this disease condition in her family. On physical examination of the patient, oozing of the fluid from the lesion was noted. Histopathological examination of the skin biopsy revealed angiofibrolipoma; after confirming the patient's diagnosis, the patient was referred to the surgery department for further management.

**Figure 1:**
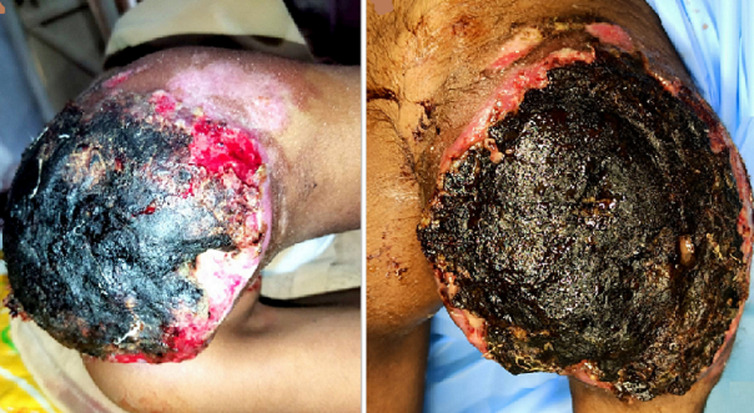
right gluteal capillary hemangioma

